# Supplementary figures and images for: Genomes of Gut Bacteria from Nasonia Wasps Shed Light on Phylosymbiosis and Microbe-Assisted Hybrid Breakdown
Source: mSystems. 2021 Apr 6;6(2):e01342-20. doi: 10.1128/mSystems.01342-20 (PMC8547009; doi:10.1128/mSystems.01342-20)

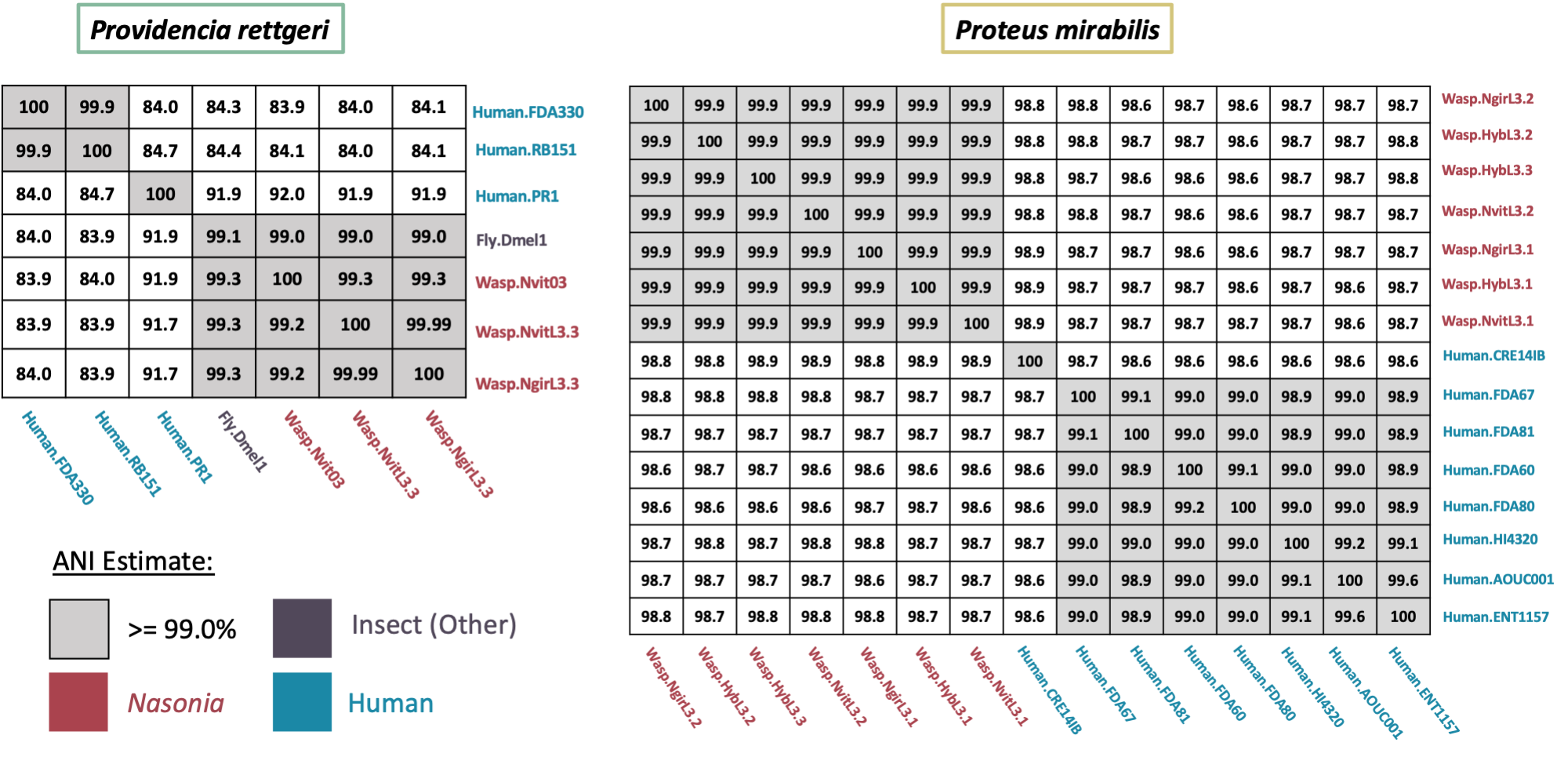

Supplement: FIG S1 [file msystems.01342-20-sf001.tif]

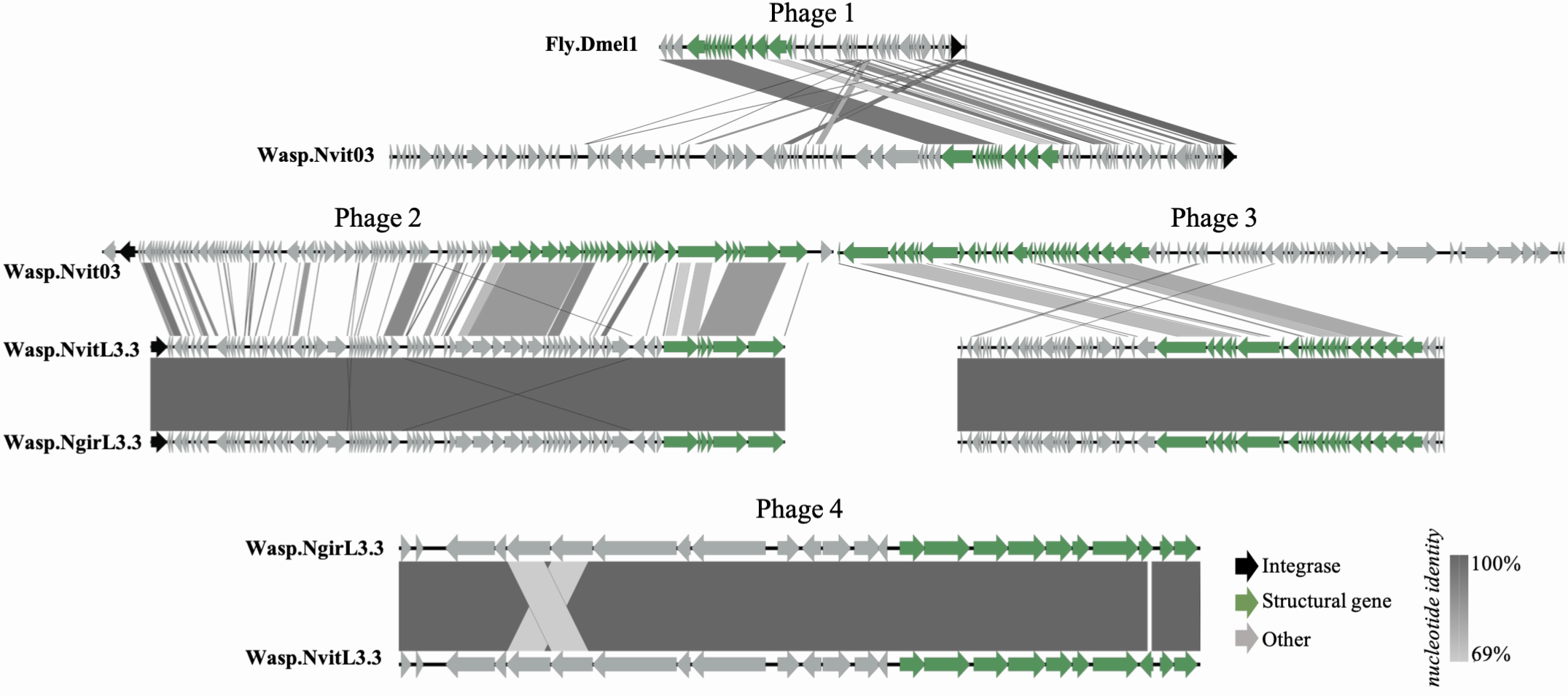

Supplement: FIG S2 [file msystems.01342-20-sf002.tif]

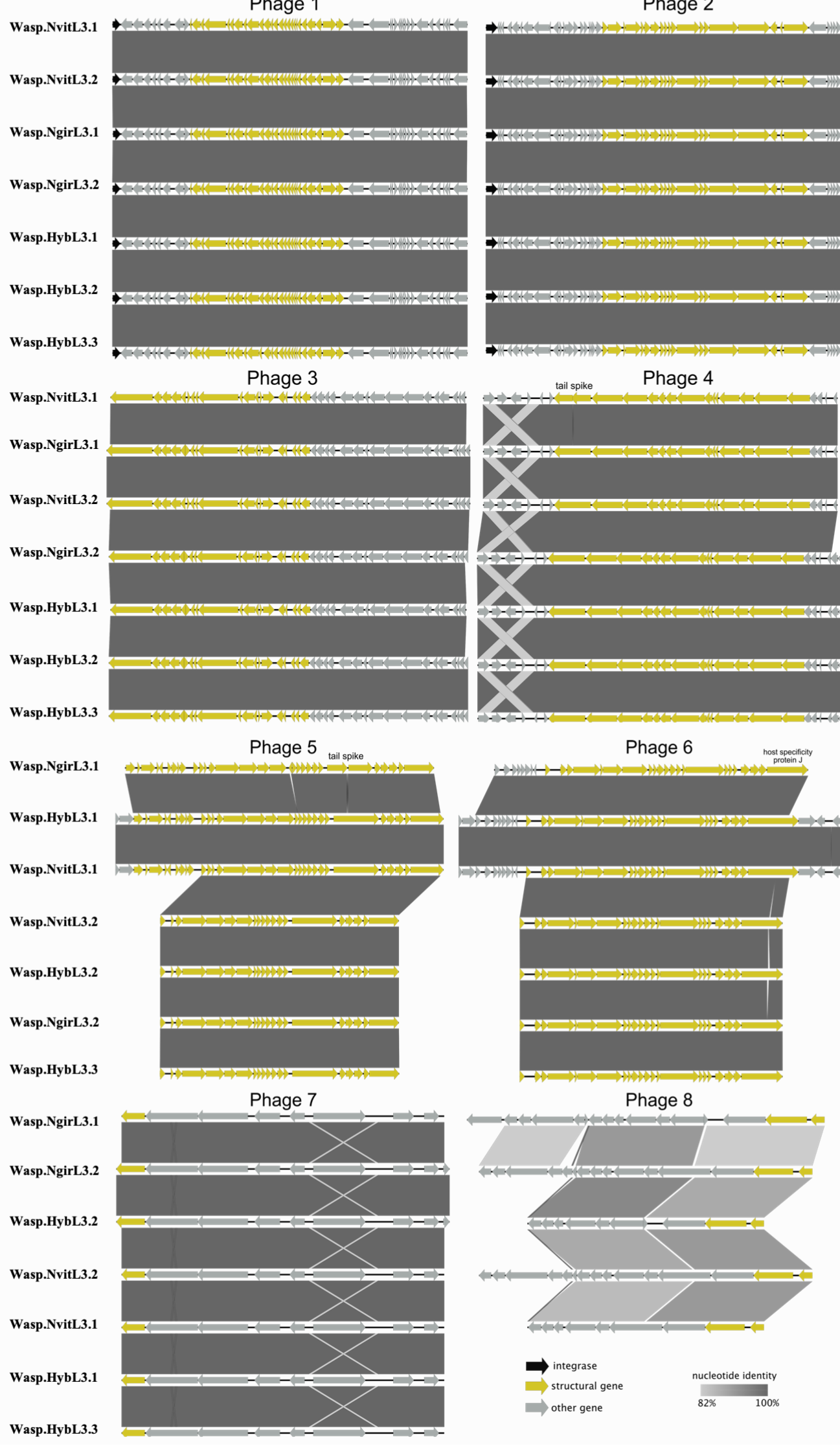

Supplement: FIG S3 [file msystems.01342-20-sf003.tif]
